# Immunology of Stress: A Review Article

**DOI:** 10.3390/jcm13216394

**Published:** 2024-10-25

**Authors:** Amna Alotiby

**Affiliations:** Department of Hematology and Immunology, Faculty of Medicine, Umm Al-Qura University, Makkah 24381, Saudi Arabia; aamogaty@uqu.edu.sa

**Keywords:** immunology of stress, promoting immune health, stress–immune system relationship, acute vs. chronic stress effects, stress management and immunity

## Abstract

Stress significantly impacts the immune system, affecting susceptibility to illness and overall health. This review examines the intricate relationship between stress and the immune system, offering insights having practical implications for health and disease prevention. Stress can significantly trigger molecular and immune modulation, affecting the distribution and trafficking of immune cells in various organs and altering their composition in the blood. The review delves into two key pathways connecting stress and immunity: the hypothalamic–pituitary–adrenal (HPA) axis and the sympathetic nervous system. Stress activates the neuroendocrine system and triggers microglia in the brain, releasing stress hormones and neurotransmitters that modulate the function and movement of immune cells. Acute stress can temporarily strengthen immunity and promote protection during infection; in contrast, chronic stress dysregulates or inhibits immune functions. Chronic stress causes an increase in cortisol levels through the HPA axis, ultimately suppressing the immune response. Recognizing stress triggers and implementing effective stress management techniques can significantly impact individuals’ well-being. This review indicates that immune cells express genes differentially in response to stress, suggesting individual variabilities in the immune response against stress. This underscores the need for a personalized approach to stress management. This review also highlights the potential link between chronic stress and autoimmune disorders and warrants further investigation.

## 1. Introduction

Stress is induced by a difficult situation or a challenging incident, known as a stressor, to which the body develops either a physiological or psychological response called the stress response. When the body is under stress, the immune system actively responds by mobilizing specific cells into the bloodstream, which helps in fight or flight. Blood levels of pro-inflammatory cytokines increase during stress, thus activating latent viruses [1]. Selye, the “father” of stress, described the three phases of stress. The first and second phases are alarm reactions and resistance, respectively, and are not dangerous. The third phase of stress (exhaustion) can cause health deterioration. Stress influences the body through primary and secondary lymphoid tissues via sympathetic fibers in the brain. These fibers release substances that control the immune response and bind to white blood cells [2,3]. Lymphocytes exhibit stress-dependent adrenergic receptors that alter their activity due to adrenergic stimulation [4].

Additionally, behavioral patterns are linked to stress pathways and the immune system. Several published reviews have examined the impact of stress on the overall health of the immune system and its role in increasing the likelihood of infection or disease [4,5,6,7,8]. However, none of these reviews have suggested stress as a trigger or stimulator of the immune response, which is known as the immunology of stress. This comprehensive review fills that gap, encompassing the response of immune cells to stress as a trigger and the pathways that connect stress to the immune system. It also explains the physiological and physicochemical responses to stress, the impact of acute and chronic stress on the immune system, and the molecular and immune modulation induced by stress.

## 2. Immune System Overview

The immune system is a highly intricate and dynamic network responsible for defending the body against a diverse array of pathogens, including bacteria, viruses, fungi, and parasites. It can be categorized into two primary immune responses: innate and adaptive immunity, each characterized by distinct cellular components and mechanisms [9].

The innate immune response serves as the first line of defense, providing immediate but non-specific protection. Key players in this response include macrophages, neutrophils, dendritic cells, natural killer (NK) cells, and mast cells. Macrophages, derived from monocytes, are pivotal in engulfing pathogens and dead cells through phagocytosis while also releasing pro-inflammatory cytokines like interleukin-1 (IL-1), IL-6, and tumor necrosis factor-alpha (TNF-α) to recruit additional immune cells to sites of infection [10]. Neutrophils, the most abundant white blood cells, respond rapidly to infections by phagocytosis, releasing antimicrobial substances from granules and using neutrophil extracellular traps (NETs) to capture pathogens [11]. Dendritic cells act as crucial messengers between innate and adaptive immunity by capturing antigens and presenting them to T cells, thereby initiating the adaptive immune response [12]. Natural killer (NK) cells play an essential role in identifying and eliminating virus-infected or malignant cells through the release of perforins and granzymes, inducing apoptosis [13]. Mast cells are involved in allergic responses and defense against parasites, releasing histamine and inflammatory mediators upon activation [14].

In contrast, the adaptive immune response is characterized by its specificity and memory, providing long-lasting immunity. This response involves B and T cells, which are critical for orchestrating effective immune responses. Upon encountering specific antigens, B cells activate and differentiate into plasma cells that secrete antibodies and memory B cells that ensure rapid responses upon subsequent exposures [15]. T cells are divided into helper T cells (CD4+ T cells) and cytotoxic T cells (CD8+ T cells). Helper T cells coordinate the immune response by releasing cytokines that activate B cells and cytotoxic T cells while enhancing macrophages’ function [9]. Cytotoxic T cells directly target and kill infected or cancerous cells by recognizing specific antigens presented on major histocompatibility complex (MHC) class I molecules [16].

The immune system’s ability to distinguish between self and non-self and its capacity to remember previous encounters with pathogens is crucial for adequate protection against diseases. Maintaining immune homeostasis is crucial for the immune system to function optimally, ensuring that responses to pathogens are appropriately calibrated without triggering excessive inflammation or autoimmunity. Stress can disrupt this delicate balance, leading to a state of immune dysregulation. Elevated levels of stress hormones, particularly cortisol, can suppress the activity of key immune cells and skew cytokine production, resulting in a weakened immune response [17]. This disruption can lead to decreased production of antibodies and impaired T cell function, ultimately compromising the body’s ability to fight infections and maintain overall health [2]. Furthermore, stress can induce changes in the distribution and behavior of immune cells, such as a reduction in circulating T cells and natural killer cells, which are vital for recognizing and eliminating infected or cancerous cells. This shift can diminish immune surveillance and increase susceptibility to both infectious diseases and chronic inflammatory conditions [18].

## 3. Physiological and Physicochemical Responses to Stress

Humans react to stress via physiological mechanisms involving the sympathetic adrenomedullary and hypothalamic–pituitary–adrenal (HPA) systems. These pathways prime the body for danger by increasing cardiac output, mobilizing energy, and modifying the immunological response [19]. Extended or dysregulated stress reactions may increase the allostatic load and the risk of infections, heart disease, and hypertension. Chronic stress exposure is common in the current lifestyle and has an impact on mental health in addition to potentially exacerbating illnesses such as diabetes, obesity, cancer, and cardiovascular disease (CVD). The catabolic effects of cortisol and stress on the brain emphasize the importance of stress management for general well-being [20].

Additionally, stress can be induced by exposure to pollution. Pollutants regulate stress responses by affecting biological systems. The HPA axis of the endocrine system primarily regulates the stress response. Test performance is affected by test anxiety, which releases cortisol and activates the HPA axis. Factors such as information delivery, past knowledge, and emotional state influence stress response and nerve responsiveness during testing [21]. In neurobiology, the allopregnanolone and HPA axes play important roles in major depressive disorder (MDD) and post-traumatic stress disorder (PTSD). Severe stress exposure can alter these levels by decreasing allopregnanolone levels, thereby inducing PTSD and MDD. Changes in cortisol levels are observed in the HPA axis, which results in an increase in PTSD and MDD [22]. Athletes often experience exhaustion, mood swings, poor performance, and gastrointestinal problems during training and competitions. These effects may be linked to the release of inflammatory cytokines, microbiological chemicals, and stress hormones induced by vigorous exercise. Behavior, metabolism, and immune system regulation are all greatly influenced by the gut-brain axis, which is mediated by the gut microbiota, gut barrier, and central nervous system. Hence, maintaining a balanced diet and a stress-free attitude can be a potentially favorable approach in this regard [23].

Stress often changes the psychological functioning of the brain. The relationship between the autonomic nervous system (ANS) and neuroendocrine (NE) reactions to stress and the systems that contribute to the onset of CVD has been studied. The parameters evaluated to elucidate this association include an increase in reaction magnitudes, delayed recovery of responses, and a relative absence of stress response. Although attenuated cardiovascular responses to stress are seldom associated with CVD, they are linked to specific behavioral risk factors for CVD, such as certain health habits and depression [24].

## 4. Pathways Connecting Stress to Immune Function

The HPA axis plays a central role in the body’s response to stress. When exposed to a stressor, the hypothalamus releases corticotropin-releasing hormone (CRH), which stimulates the anterior pituitary gland to secrete adrenocorticotropic hormone (ACTH). This prompts the adrenal cortex to produce cortisol, a glucocorticoid critical for modulating immune responses [25,26]. Cortisol has a dual effect on immune function. In the short term, it enhances the activity of specific immune cells, such as NK cells, and promotes the production of pro-inflammatory cytokines, including IL-6 and TNF-α [27,28]. However, chronic exposure to high cortisol levels can lead to immune dysregulation and immunosuppression. Prolonged cortisol exposure reduces T cell proliferation and activity, diminishing the body’s ability to mount effective immune responses [29]. This immunosuppressive effect is well-documented, particularly in cases of chronic stress. For example, Zhang et al. (2020) found that high cortisol levels were linked to decreased T cell activation and proliferation in patients with chronic stress, emphasizing how elevated glucocorticoids can impair adaptive immune responses, increasing vulnerability to infections and reducing vaccine efficacy [30]. Furthermore, a recent study found that individuals with chronic insomnia showed dysregulation of the HPA axis, characterized by elevated cortisol levels and impaired immune responses, specifically demonstrating decreased lymphocyte proliferation in response to vaccination, highlighting the impact of chronic stress on immune competence [31].

In addition to the HPA axis, the sympathetic nervous system (SNS) plays a crucial role in the stress response. Activation of the SNS leads to the release of catecholamines, including adrenaline and noradrenaline, which can directly affect immune cell function [32]. These neurotransmitters have a dual effect that can modulate immune responses in a context-dependent manner. They can enhance the activity of innate immune cells such as NK cells and macrophages but may inhibit adaptive immunity by altering the function of T cells and B cells [33]. Short-term increases in catecholamines can stimulate the mobilization of immune cells from the bone marrow and increase their responsiveness [34]. However, chronic SNS activation can lead to a dysregulated immune response characterized by impaired T cell function. Prolonged catecholamine exposure has been shown to downregulate the expression of CD4+ and CD8+ T cell receptors, leading to decreased T cell activation and proliferation. This dysregulation contributes to a weakened adaptive immune response with significant health implications [3,29].

**Figure 1:**
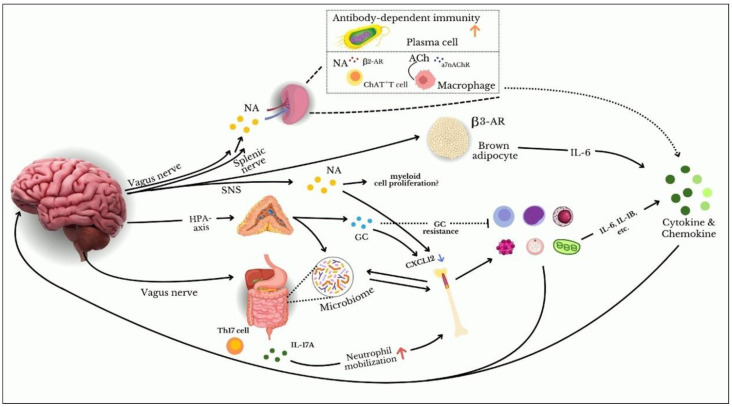
Pathways connecting stress to immune function. Stress activates the HPA axis and SNA, releasing catecholamines (noradrenaline) and glucocorticoids. These hormones alter immune cell behavior, increasing inflammatory cytokines and redistributing immune cells. This has effects on organs like the spleen and gastrointestinal tract, potentially influencing the microbiome. Abbreviations: IL: interleukin; Th17: T helper lymphocyte 17; CXCL12: homeostatic chemokine ligand; β2/β3-AR: β2/β3-adrenoceptors; Ach: acetylcholine; α7nAChR: α7 nicotinic acetylcholine receptor; HPA: hypothalamic–pituitary–adrenal axis; SNS: sympathetic nervous system; NA: noradrenaline; GC: glucocorticoids. Adapted from Ishikawa and Furuyashiki (2022) [36].

A study examined the effects of acute stress on immune function in mice. It found that acute stress led to increased norepinephrine levels, which enhanced macrophage activity but concurrently suppressed the activation of T cells. In contrast, chronic stress in mice led to decreased B cell function and impaired antibody responses, emphasizing the long-term impact of stress on humoral immunity. The findings suggest that stress-induced catecholamine release may inhibit the differentiation of B cells, thereby reducing the production of antibodies [35]. Figure 1 summarizes the above explanation of how stress influences immune function by activating the HPA axis and SNS. This activation triggers the release of catecholamines (noradrenaline) and glucocorticoids, which modulate the activity and movement of immune cells, including macrophages, NK cells, neutrophils, T cells, and B cells. The resulting changes lead to increased production of inflammatory cytokines (like IL-6 and IL-1B) and a shift in immune cell distribution. These effects can influence key organs, such as the spleen and gastrointestinal tract, and may also impact the microbiome.

## 5. Immune Response to Stress

**Figure 2:**
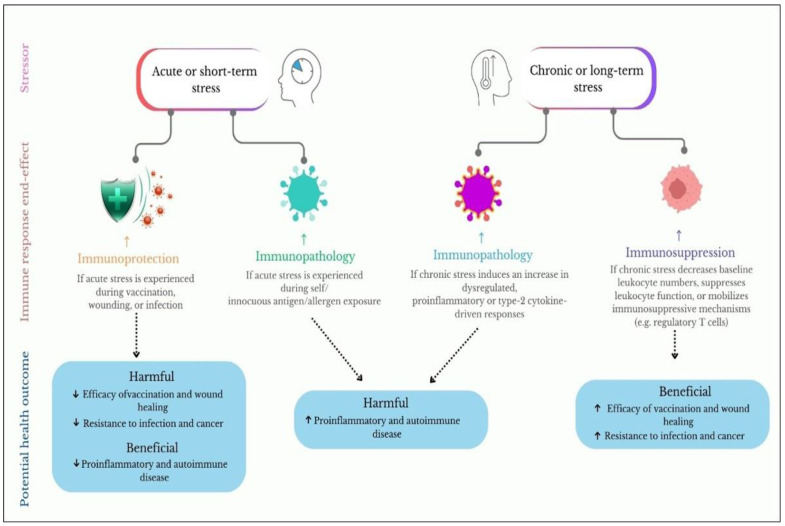
Effects of acute and chronic stress on the immune system. Acute stress temporarily enhances immune responses by activating immune cells, while chronic stress suppresses immune function, leading to increased inflammation and greater susceptibility to illness.

The immune system responds to stress through various mechanisms that can yield both beneficial and detrimental outcomes, depending on the duration and intensity of the stressor. In a controlled study by Barrett et al. (2021), researchers exposed mice to both short-term (acute) and long-term (chronic) stressors. While acute stress enhanced T cell proliferation and increased inflammatory markers, chronic stress significantly reduced cell activity and overall immune function, as seen in Figure 2. This finding highlights the importance of considering stress duration when evaluating its effects on immunity [4,33]. 

## 6. Acute Stress and Immune Activation

Acute stress refers to immediate responses to perceived threats, activating the body’s “fight or flight” response. This activation is characterized by a rapid increase in stress hormones, particularly cortisol and adrenaline, which can be beneficial in the short-term [37]. Acute stress often leads to a temporary enhancement of immune function, known as the “stress-induced immune response”, preparing the body to respond effectively to immediate threats. It facilitates the mobilization of immune cells and increases the production of pro-inflammatory cytokines. A study by Dhabhar (2014) demonstrated that acute stress enhances the mobilization of leukocytes, particularly NK cells, and neutrophils, into circulation, thereby improving immune surveillance, which is essential for early defense against infections and tumors. The research also indicated a significant increase in pro-inflammatory cytokines, such as IL-6 and TNF-α, following acute stress exposure, which is crucial for necessary inflammatory processes during physical injuries or infections [37]. Clinical studies support these findings. For instance, Cohen et al. (2007) found that individuals exposed to acute psychological stress, such as public speaking or exam-related pressure, exhibited increased levels of circulating pro-inflammatory cytokines and heightened NK cell activity [18]. Additionally, a study by Zhang et al. (2020) assessed the immune response of participants undergoing acute psychological stress, showing a significant increase in circulating pro-inflammatory cytokines (IL-6, TNF-α) and enhanced NK cell activity immediately following the stressor [30]. These findings suggest that acute stress may prime the immune system to respond rapidly to potential threats.

## 7. Chronic Stress and Immune Dysregulation

In contrast, chronic stress involves prolonged exposure to stressors and is associated with detrimental health effects, particularly regarding cytokine production. Chronic stress has a dual impact on cytokine levels, simultaneously increasing both pro-inflammatory and anti-inflammatory cytokines [29].

Chronic stress often leads to elevated levels of pro-inflammatory cytokines, such as IL-6, TNF-α, and IL-1. The activation of the HPA axis under stress conditions results in the release of glucocorticoids, primarily cortisol. While cortisol initially dampens inflammatory responses, prolonged exposure can paradoxically increase pro-inflammatory cytokine production [28,38]. Research indicates that stress can induce a state of immune activation, where immune cells like macrophages and T cells become hyper-responsive, driven by stress-related neurotransmitters like norepinephrine, which can enhance the secretion of pro-inflammatory cytokines [17]. Furthermore, studies suggest chronic stress may alter gene expression cytokine signaling, contributing to a sustained inflammatory state [39]. The chronic elevation of pro-inflammatory cytokines is linked to various health issues, including cardiovascular disease, metabolic syndrome, and mental health disorders such as depression and anxiety [25,40]. A study found that individuals experiencing chronic stress exhibited significantly higher levels of IL-6, correlating with increased risk factors for cardiovascular diseases. This chronic stress is linked to heightened arterial stiffness and endothelial dysfunction, both critical contributors to cardiovascular disease development. Furthermore, the persistent inflammatory state associated with stress can exacerbate these physiological changes, as stress-induced inflammation may damage blood vessels and promote atherosclerosis [29].

Chronic stress can also lead to increased levels of anti-inflammatory cytokines, such as interleukin-10 (IL-10) and transforming growth factor-beta (TGF-β). This response may serve as a compensatory mechanism to counterbalance the effects of pro-inflammatory cytokines [8]. However, while elevated levels of anti-inflammatory cytokines may seem beneficial, prolonged stress can disrupt the balance between pro-inflammatory and anti-inflammatory responses. Evidence suggests that the chronic elevation of both types of cytokines may lead to immune dysregulation. Elevated anti-inflammatory cytokines may not be sufficient to counteract the effects of sustained pro-inflammatory cytokines, resulting in immune suppression or developing autoimmune disorders [8,41]. Over time, the responsiveness of immune cells to cortisol diminishes, leading to a decrease in the expression of cortisol receptors. This decline results in reduced anti-inflammatory effects of cortisol, thereby fostering a state of chronic inflammation. This chronic stress triggers immune dysregulation, contributing to the onset and flare-ups of various autoimmune conditions [41]. For example, studies involving patients with rheumatoid arthritis indicate that individuals with elevated stress levels report increased disease activity and more frequent flare-ups. Stress management techniques, such as cognitive behavioral therapy, have been shown to alleviate disease severity, demonstrating that psychological interventions can significantly impact physical health [42].

Chronic stress can also lead to decreased T and B lymphocyte proliferation, impairing/suppressing the adaptive immune response and increasing susceptibility to infections [33,38]. For example, a study on individuals with chronic illness found that those experiencing ongoing stress had significantly lower T-cell counts and antibody responses to influenza vaccination than those with lower stress levels [35]. Cohen et al. (2013) also found that individuals under high chronic stress conditions were more prone to developing upper respiratory infections [18]. The study linked this increased susceptibility to impaired immune responses, emphasizing the clinical relevance of stress management.

The relationship between chronic stress and cytokine production is dynamic and multifaceted. Individual differences, such as genetic predispositions, psychological resilience, and environmental factors, can significantly influence this balance. Studies have shown that individuals with adaptive coping mechanisms tend to have a more favorable cytokine profile, suggesting that effective stress management strategies can mitigate the adverse effects of chronic stress [41,43,44].

## 8. Stress-Induced Molecular and Immune Modulations

Stress causes molecular and immunological modulations, altering immune function and response to infection and disease. Severe stress can create an anergic state in the immune system, reducing protective immunity and increasing vulnerability to infection [45]. Stress can activate signaling pathways that affect the expression of molecules involved in immunity [44]. This allows the immune system to recognize stressed cells. Stress may additionally trigger immunogenic cell death, which helps the immune system recognize and kill cancer cells [46]. Stress can alter the innate immune mechanisms by altering the activation status of myeloid lineage cells and increasing the synthesis and release of inflammatory proteins. These stress-induced immunological and biochemical alterations potentially affect vaccine efficacy, therapeutic strategies, and overall health and well-being [29,38].

Various mechanisms modulate immune responses via stress-induced transcription factors. One approach is the activation of redox-sensitive transcription factors and enzymes, resulting in the generation of reactive oxygen and nitrogen species, along with altered intracellular antioxidant levels [47]. Another approach is controlling the immunological transcriptome through intracellular signaling pathways, including the phosphatidylinositol 3-kinase or protein kinase B signaling pathway, mitogen-activated protein kinases, and calcium signaling [48]. Immunological responses can also be modulated by changes in histones, noncoding RNAs, transcriptional regulators, and DNA methylation [49]. Furthermore, CRH is required for stress-induced gene expression in immune cells, such as splenocytes. Collectively, these mechanisms help stress-induced transcription factors modulate immunological responses [50].

Acute and chronic stress are associated with increased levels of pro-inflammatory cytokines, including TNF-α, IL-1β, and IL-6 [8,17]. Furthermore, stress-induced neuroimmune priming has been observed, indicating a history of stress-sensitizing the immune system and enhanced pro-inflammatory effects [5]. The immune system can be activated and triggered by stress, leading to the release of pro-inflammatory cytokines. This leads to exacerbation of inflammatory conditions. The stress-induced p38 mitogen-activated protein kinase signaling pathway, in turn, activates transcription factors such as the cAMP-response element binding protein and c-Jun, which attach to interferon-stimulated genes present in macrophages [47]. The transcriptional factor, nuclear factor kappa B (NF-κB), is associated with stress-induced immunological regulation. Stress may increase the binding activity of NF-κB DNA in peripheral blood mononuclear cells, resulting in increased mRNA expression of the pro-inflammatory cytokines IL-6 and IL-1β [39]. The NF-κB family includes NF-κB1, NF-κB2, RELB, RELA, and interferon-regulatory factors such as IRF1, IRF5, IRF7, and IRF8, which are significant transcription factors involved with immune response genes and are inhibited in leukocytes during battlefield-like stress [39]. Other stress-activated genes associated with immunological regulation include TRP53INP1, MKP1, Rtp801, Gilz, and BNIP3. In response to stress, immune cells express these genes differentially, which is affected by the type and duration of stress [51].

## 9. Conclusions and Future Directions

The immunology of stress represents a critical intersection between stress and the immune system, with acute and chronic stressors exerting distinct effects on immune function. Acute stress activates immune cells and has short-term positive effects. However, chronic stress has been shown to significantly disrupt immune function through mechanisms such as the activation of the HPA axis and the SNS, alterations in cytokine profiles, and modifications in immune cell dynamics. These disruptions not only heighten susceptibility to infections and exacerbate autoimmune conditions but may also influence the progression of cardiovascular diseases and various other health outcomes.

Understanding these complex interactions underscores the urgent need for integrated approaches that simultaneously address psychological and immunological health. Future research should prioritize mechanistic studies, intervention trials, and longitudinal analyses to enhance our understanding of the intricate relationship between stress and immune function. However, this review has limitations: it does not specify the duration of chronic stress associated with autoimmunity or reduced vaccination efficacy nor explores potential variations in stress responses based on gender and age. Additionally, it lacks a discussion of specific interventions or therapeutic approaches crucial for developing effective strategies. Further research is recommended to investigate how autoimmune disorders and vaccination efficacy are affected by long-term stress. Furthermore, addressing the disparities in immune regulation and stress response based on gender and age is important. The development of individualized stress reduction plans based on personal characteristics needs to be designed.

Moreover, public health initiatives to reduce stress and promote mental well-being can significantly bolster community resilience and overall health. By adopting a holistic perspective that encompasses psychological, biological, and social dimensions, we can formulate effective strategies to mitigate the adverse effects of stress and optimize immune system function. As our understanding of this field continues to evolve, so will our capacity to foster healthier and more resilient populations.
